# Predicting cognitive decline in Parkinson’s disease using FDG-PET–based supervised learning

**DOI:** 10.1172/JCI157074

**Published:** 2022-10-17

**Authors:** Samuel Booth, Kye Won Park, Chong Sik Lee, Ji Hyun Ko

**Affiliations:** 1 Department of Human Anatomy and Cell Science, Max Rady College of Medicine, Rady Faculty of Health Sciences, University of Manitoba, Winnipeg, Manitoba, Canada.; 2 Neuroscience Research Program, Kleysen Institute for Advanced Medicine, Health Sciences Centre, Winnipeg, Manitoba, Canada.; 3 Department of Neurology, Asan Medical Center, University of Ulsan College of Medicine, Seoul, South Korea.

**Keywords:** Neuroscience, Dementia, Glucose metabolism, Parkinson disease

## Abstract

**Background:**

Cognitive impairment is a common symptom of Parkinson’s disease (PD) that increases in risk and severity as the disease progresses. An accurate prediction of the risk of progression from the mild cognitive impairment (MCI) stage to the dementia (PDD) stage is an unmet clinical need.

**Methods:**

We investigated the use of a supervised learning algorithm called the support vector machine (SVM) to retrospectively stratify patients on the basis of brain fluorodeoxyglucose-PET (FDG-PET) scans. Of 43 patients with PD-MCI according to the baseline scan, 23 progressed to PDD within a 5-year period, whereas 20 maintained stable MCI. The baseline scans were used to train a model, which separated patients identified as PDD converters versus those with stable MCI with 95% sensitivity and 91% specificity.

**Results:**

In an independent validation data set of 19 patients, the AUC was 0.73, with 67% sensitivity and 80% specificity. The SVM model was topographically characterized by hypometabolism in the temporal and parietal lobes and hypermetabolism in the anterior cingulum and putamen and the insular, mesiotemporal, and postcentral gyri. The performance of the SVM model was further tested on 2 additional data sets, which confirmed that the model was also sensitive to later-stage PDD (17 of 19 patients; 89% sensitivity) and dementia with Lewy bodies (DLB) (16 of 17 patients; 94% sensitivity), but not to normal cognition PD (2 of 17 patients). Finally, anti-PD medication status did not change the SVM classification of the other set of 10 patients with PD who were scanned twice, ON and OFF medication.

**Conclusions:**

These results potentially indicate that the proposed FDG-PET–based SVM classifier has utility for providing an accurate prognosis of dementia development in patients with PD-MCI.

## Introduction

Cognitive impairment is a frequent manifestation of Parkinson’s disease (PD), with dementia developing in approximately 50% of patients within 10 years of diagnosis (1), resulting in a decrease in the patient’s quality of life and an increase in the societal and economic burden (2). One of the greatest risk factors for dementia in PD is mild cognitive impairment (MCI), in which the patient performs 1 to 2 standard deviations (SDs) below normal in at least 2 cognitive domains (or in 2 different tests within a single domain), but the patient’s daily functioning is not significantly affected (3). Parkinson’s disease with dementia (PDD) is the stage at which the cognitive deficits are present in multiple cognitive domains and the symptoms interfere with the patient’s normal social, occupational, or personal care functioning independent of motor impairment (4). Related to PDD is dementia with Lewy bodies (DLB), a synucleopathy that has substantial phenotypic overlap with PDD. A differential diagnosis of DLB is based on whether cognitive impairment precedes or begins within 1 year of the onset of parkinsonian motor signs, whereas PDD is diagnosed when dementia occurs within the context of established PD (5). DLB is considered part of the same disease spectrum as PD, with important common underlying pathological mechanisms, while at the same time differing in neurochemical, morphological, and symptom characteristics (6).

Approximately 90% of patients with PD-MCI progress to PDD eventually, but some remain stable or even revert to a cognitively normal state (7–10). Certain clinical characteristics such as poor performance on balance and gait assessment, deficits in attention and verbal memory, smell dysfunction, and sleep problems are often associated with an accelerating cognitive decline in patients with PD-MCI (7, 11–14). A meta-analysis suggested that age, male sex, greater severity of motor symptoms, hallucination, REM sleep behavior disorder, smoking, and hypertension increase the risk of PDD (15), whereas a recent longitudinal study suggested that baseline neuropsychological and clinical characteristics do not robustly predict further cognitive decline in patients with PD-MCI, meaning that accurate prognosis is an unmet challenge (16).

Brain imaging techniques have the potential to show changes in brain function that occur in patients in the early stages of PDD and can thus be used as a predictive biomarker. Previous cross-sectional studies have identified several brain regions where metabolic reductions detected by fluorodeoxyglucose-PET (FDG-PET) are associated with PDD. Compared with patients with PD-MCI, those with PDD have been shown to have metabolic reductions in the inferior (17) and lateral (18) frontal regions, as well as in the parietal (17–20), temporal (21), and occipital lobes (22) and in the hippocampal gyrus and the amygdala (20, 23). In several of these studies, hypometabolism in the occipital-parietal regions was shown to differentiate patients with PDD and those with PD-MCI (18, 24–27).

Longitudinal studies largely support the idea that posterior cortical hypometabolism heralds a rapid cognitive decline in PD (27–29). At baseline, PDD converters (nondemented patients with PD who later developed PDD) showed significant parietal/occipital hypometabolism (27) with marked longitudinal reductions in the cingulate and precuneus (23, 24). However, the high heterogeneity of results as well as large interindividual variability have limited the clinical translation of FDG-PET as a prognostic tool for PDD development (30, 31).

Recently, there has been increasing interest in machine learning techniques that can predict diagnosis, prognosis, and treatment from brain imaging data at an individual level (32, 33). The advantage of machine learning is that it can discover specific differential patterns that may not be apparent a priori. For example, the support vector machine (SVM) utilizes “kernel trick” to build a hyperdimensional space, whereby a hyperplane is drawn to divide the data sets into 2 predefined groups. The inner product between the hyperplane and a prospective data element (e.g., FDG-PET scan of a patient whose scan was not used to train the original SVM) determines the group designation of the prospective patient. Previously, we have shown that the SVM-based classification technique performed better at predicting future cognitive decline from FDG-PET scans of patients with prodromal Alzheimer’s disease (AD) (patients with MCI who later developed AD compared with those who did not) than did general linear model– or principal component analysis–based techniques (34).

A recent systematic review of 86 studies using different machine learning techniques in AD found that, while the classification performance was strong at identifying demented versus normal controls, separating MCI from normal controls and predicting further cognitive decline at the MCI stage was much more challenging (35). Fewer studies have been performed stratifying patients with PD on the basis of cognitive status using machine learning models, although some success has been reported using gray matter volume (36). Additionally, a recent study using a supervised learning algorithm showed that patients with PD-MCI and cognitively normal patients with PD (PDNC) could be discriminated on the basis of functional connectomics (37). However, there is still limited research on predicting future cognitive decline using baseline neuroimages.

Our aim was to develop a metabolic pattern that predicts future dementia development in patients with PD-MCI. We used FDG-PET data from 43 patients with PD-MCI, who were followed for a period of up to 8 years. Twenty-three of the patients progressed to PDD within this time frame. An SVM classifier was developed to predict future cognitive decline using baseline FDG-PET scans.

## Results

**Figure 1:**
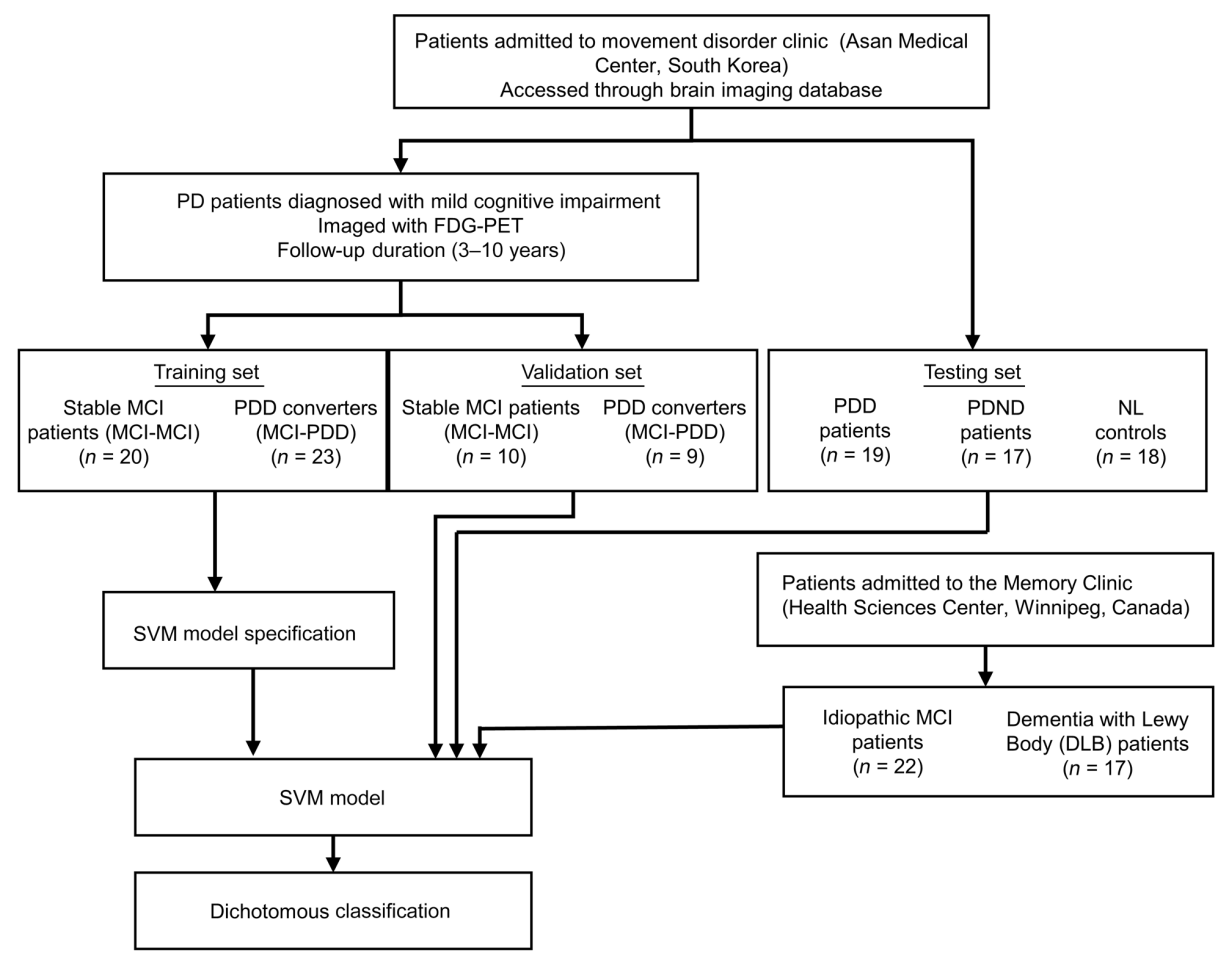
Flowchart of the study design and participating patients from the Asan Medical Center in Seoul, South Korea, and the Health Sciences Center in Winnipeg, Canada.

**Figure 2:**
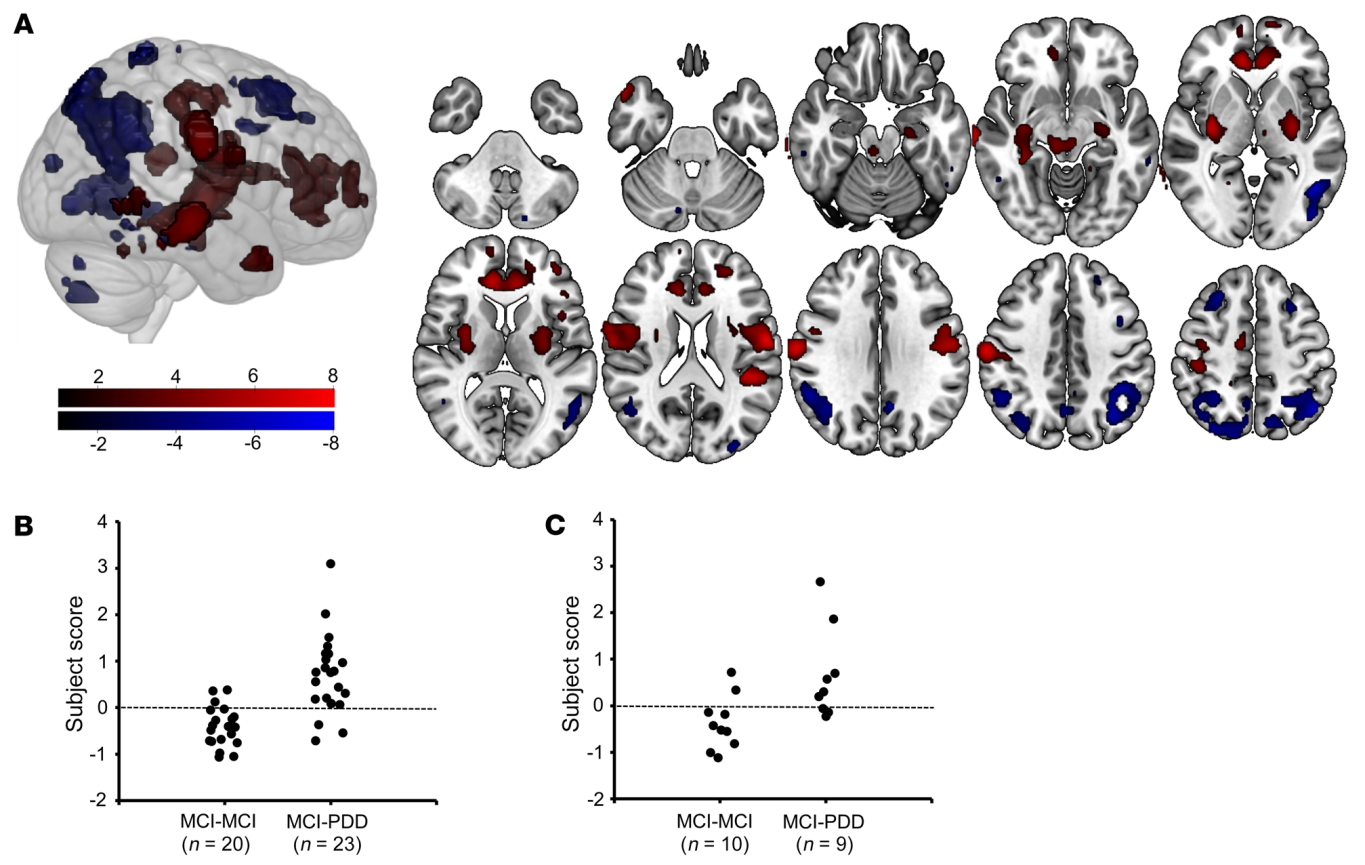
PDDCP, a metabolic pattern identified with SVM, is associated with the prediction of transition from MCI to dementia in patients with PD. (**A**) Anatomical map of the linear predictor coefficients for MCI-PDD generated by the classification machine. The coefficient map is thresholded at *P <* 0.05 as defined by a permutation test, so that only regions at a significance level of *P <* 0.05 are shown. Increased metabolism in the red regions and decreased metabolism in the blue regions are associated with transition from MCI to dementia in patients with PD. (**B**) K-fold cross-validated individual subject scores from the MCI-MCI and MCI-PDD groups in the training set. Positive subject scores were classified as MCI-PDD, while negative subject scores were classified as MCI-MCI, resulting in 86.96% sensitivity and 85.00% specificity. (**C**) Individual subject scores from the MCI-MCI and MCI-PDD groups in the independent set, resulting in 67% sensitivity and 80% specificity.

A flowchart outlining the study design and participant groups can be found in Figure 1. The raw demographic and clinical variables for each patient cohort used in this study are available in Supplementary Table 1. In the training data, there were no significant differences between age at diagnosis, disease duration, or Mini-Mental State Examination (MMSE) or the Movement Disorder Society–Sponsored Revision of the Unified Parkinson’s Disease Rating Scale (MDS-UPDRS) scores. Within the training set, the MCI-PDD converters scored significantly worse on the verbal fluency domain than did the patients with stable MCI (MCI-MCI) at baseline [*t*(38) = 2.543, *P* = 0.016]. The MCI-PDD converters in the testing set data had a significantly shorter PD duration and a significantly shorter time to PDD conversion than did the patients in the training set [*t*(17) = 4.143, *P <* 0.001]. All patients had an average follow-up period of 71.145 months. The resulting SVM-based classifier predicted PDD conversion from MCI status with 95% sensitivity and 91% specificity. We confirmed the high classification performance with 10-fold cross-validation (87% sensitivity and 85% specificity; Figure 2, B and C). The classifier’s hyperplane was characterized by decreased glucose metabolism in the middle frontal gyrus, supramarginal gyrus, angular gyrus, precuneus, middle temporal lobe, and parietal lobe, and increased glucose metabolism in the anterior cingulum and putamen, insular gyrus, mesiotemporal lobe, postcentral gyrus, and supplementary motor area (SMA) (Figure 2A). The reliability of the spatial pattern of hyper- and hypometabolism was demonstrated with a 10,000-fold permutation test (*P <* 0.05). The topography of this PDD prediction pattern was distinct from the spatial metabolic pattern that predicted AD (34) when examined with the spatial similarity test correcting for autocorrelation (*r* = 0.1079, *P* = 0.509) (38). Neither the labels nor the subject scores for the PDD conversion pattern (PDDCP) were significantly different between sexes within each group (*P* > 0.4).

We evaluated the classifier’s performance in an additional testing set of 19 patients, 10 of whom had stable MCI and 9 of whom progressed to PDD. The SVM classifier predicted progression from MCI to PDD with an accuracy of 73.7% and a sensitivity and specificity of 67% and 80%, respectively (Figure 2C). The overall classification performance was not significantly different between k-fold cross-validation and the independent test samples (accuracy: χ^2^ = 1.380, *P =* 0.288; sensitivity: χ^2^ = 1.748, *P =* 0.314; specificity: χ^2^ = 0.120, *P =* 1.000).

**Figure 3:**
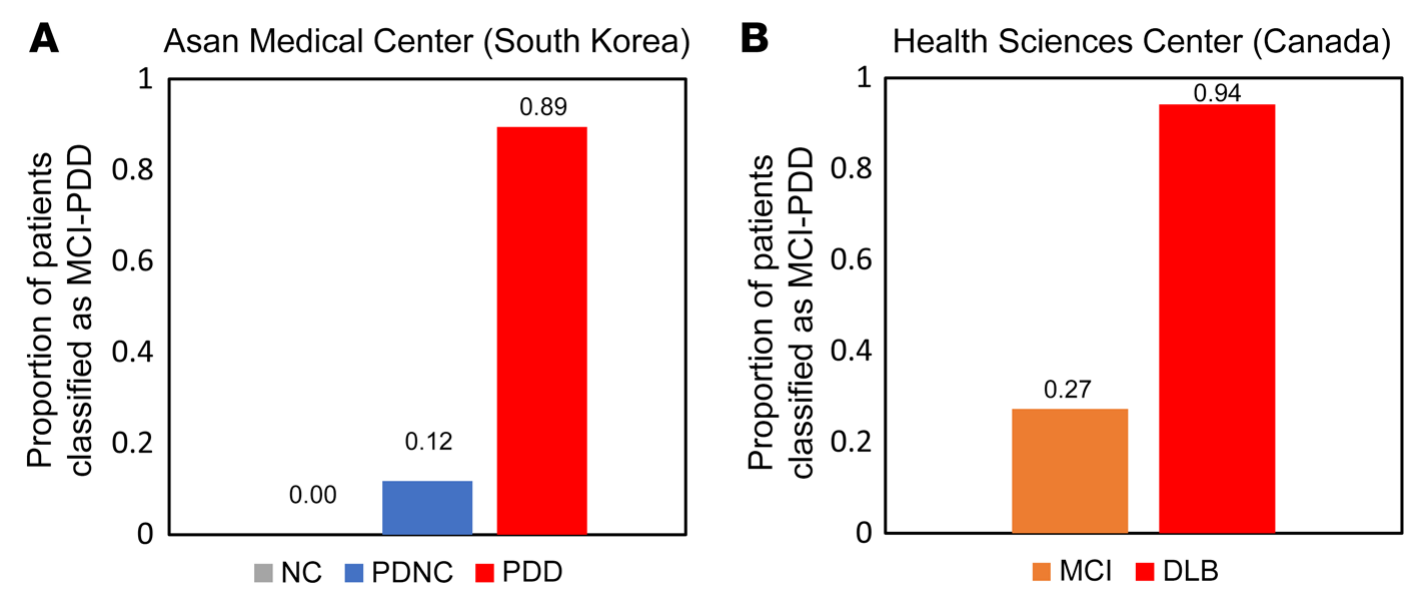
SVM classification results for validation sets. Bars show the proportion of patients classified as PDD converters by the SVM model from the additional validation set from the Asan Medical Center (**A**) using 18 normal controls (NC), 17 patients with PDNC, and 19 patients with clinically confirmed PDD. (**B**) In the validation set from the Health Sciences Center, data on 22 patients with clinically confirmed MCI and 17 with DLB were used.

To examine whether the classifier was specific only to the prodromal stage (i.e., the abnormal brain metabolic pattern expression subsides after PDD conversion), we used the classifier to assess PDD (*n* = 19), PDNC (*n* = 17), and normal controls (*n* =18) from the Asan Medical Center group. None of the normal controls were classified as PDD converters. A low percentage (11.8%) of the patients with PDNC were classified as PDD converters, despite similar disease duration and age of onset and worse UPDRS scores compared with the MCI-PDD training set data [*t*(36) = –1.551, *P* = 0.130; *t*(38) = –0.701; *P* = 0.488; *t*(27), *P* = 0.001]. A total of 89.5% of patients with clinically confirmed PDD were classified as PDD converters (Figure 3A).

We examined whether the classifier was also sensitive to patients with DLB, a synucleopathy with pathophysiology overlapping that of PD. We used the SVM model to classify patients with MCI versus those with DLB from the Health Sciences Center in Winnipeg. The SVM model classified 94.12% of patients with DLB as PDD converters, whereas 27.27% of patients with MCI were classified as PDD converters (Figure 3B).

To assess whether the pattern identified by SVM was modulated by anti-PD medication, 10 nondemented patients with PD were scanned OFF and ON their clinically determined anti-PD medication including levodopa (l-DOPA). The classifier identified the same (3 of 10) patients as PDD converters under both drug conditions. A pairwise *t* test indicated that individual SVM subject scores were not significantly different OFF or ON l-DOPA [*t*(9) = –1.069, *P* = 0.31], indicating that the subject scores (hence, the SVM-based classification) were not modulated by anti-PD medication.

## Discussion

In this study, we used the SVM to build a classifier that predicts whether patients will maintain stable MCI (mean follow-up duration: 5 years) or progress to PDD based on FDG-PET scans at baseline. The classifier yielded a PDD conversion pattern (PDDCP) that was characterized by hypometabolism in the frontal and parietal-temporal regions, as well as by an increase in metabolism in the putamen, insula, SMA, and mesiotemporal lobe. The classifier achieved good sensitivity (86.96%) and specificity (85.00%) with k-fold cross-validation. In the testing data set, the classifier predicted future development of PDD at the MCI stage with an accuracy of 74.3%. Specificity in the testing set was similar to that for the training set with k-fold cross-validation (80.0%), whereas the sensitivity in the training set was lower (67.7%), the performance of which was not significantly different from the k-fold cross-validation. The overall accuracy of 70%–80% from MCI to dementia conversion is similar to other machine learning–based neuroimaging classification models that predicted AD development from the prodromal MCI stage (39–41). For example, a recent meta-analysis by Grueso and Viejo-Sobera performed in 2021 found that the SVM was able to predict future AD progression with a mean accuracy of 75.4%, similar to what was achieved in this study, despite the availability of large multicenter data sets such as the Alzheimer’s Disease Neuroimaging Initiative (ADNI) database (39). Interestingly, sensitivity was relatively robustly preserved in the testing data set compared with specificity. The ability to screen out those patients with PD-MCI who would not progress to PDD has great utility in selecting patients for clinical trials testing novel neuroprotective or disease-modifying therapies against PDD progression, as disease heterogeneity in these studies can lead to a high variance in outcomes, making it difficult to show treatment effects (42).

Within the training set data there was no significant difference in disease duration, age of onset, or UPDRS scores between the stable MCI patients and the MCI-PDD converters, indicating that the PDD conversion–related pattern was specific to a cognitive metabolic substrate and not a general measure of disease severity comorbid with cognitive decline. Using an additional validation set of scans from patients with clinically confirmed PDD and patients with PDNC, we have also demonstrated that the PDDCP expression was not unique to the prodromal stage of PDD, as greater than 89% of patients who currently had PDD were also classified as PDD converters. Of note, 94.12% of patients with DLB were also classified as PDD converters, confirming that there were no phenotypical differences between PDD and DLB (6). On the other hand, greater than 88% of patients with PDNC who maintained normal cognition for an average of 8 years since their PD diagnosis were classified as nonconverters. Both MCI-PDD converters used in the training set and the PDNC patients had a similar disease duration and age at onset. Although we found a significant difference in UPDRS scores between these groups, symptom severity was actually worse in the patients with PDNC, which helps to support the idea that the PDD converter classification is specific to a cognitive substrate of PD–related metabolic abnormality and not simply a measure of mores severe disease progression.

Importantly, the PDDCP was not modulated by anti-PD treatment. This is important, as many FDG-PET–based neuroimaging measures in PD are sensitive to dopamine replacement therapy (43, 44). This means that PDDCP can be used for classification without requiring patients to go through medication withdrawal, which may be detrimental to imaging quality due to tremor symptoms. Additionally, withdrawal of medication is unfavorable to patients’ comfort. Insensitivity to medication means that PDDCP can be readily used in clinics. Our observation that the PDDCP is not modulated by dopaminergic therapy is in line with the observation that the majority of cognitive symptoms in PD do not appear to improve with l-DOPA.

The topology of PDDCP is broadly in line in several characteristics, similar to what has been reported in previous longitudinal studies that examined metabolic indicators of cognitive decline in PD. Recent longitudinal studies have shown that increased hypometabolism in parietal regions, including the precuneus and fusiform gyrus, is associated with worsening cognitive decline in PD, whereas prefrontal hypometabolism may indicate a cognitively stable PD-MCI subtype (26–29). Our classifier likewise showed a pattern of posterior parietal and temporal hypometabolism.

It has been shown that abnormal hypometabolism in the parietal and occipital cortices may reflect dysfunction in nondopaminergic systems in PD, particularly the cholinergic system. Considerable loss of cholinergic projections from the cholinergic basal forebrain to subcortical and neocortical regions is observed in patients with PDD (45–47). In support of this, PET-based imaging experiments have shown that the degree of cholinergic dysfunction is closely correlated to cognitive decline in patients with PD (48–53). Both neuropsychological and experimental data have suggested the dual syndrome hypothesis, in which a more rapidly progressing posterior cortical/visuospatial phenotype manifests as poor performance on visual recognition and spatial tasks and may be responsive to cholinergic therapy (54). In support of this, we found that reduced metabolism in frontal/executive regions was not a strong predictor of PDD conversion, whereas reduced metabolism in posterior parietal regions was. Additionally, the observation that this pattern was not modulated by l-DOPA treatment also supports the idea of a nondopaminergic substrate for this pattern. Interestingly, increased occipital hypometabolism, which is a hallmark of PD-associated atrophy, was not strongly represented in our model. Occipital atrophy may occur in the later stages of PD-related cognitive decline and is not itself a strong predictor of cognitive decline in the prodromal stage (55).

Our classifier also showed that several hypermetabolic regions are predictive of dementia development in patients with PD-MCI. While it is not an uncommon observation (35, 56, 57), and although these hypermetabolic regions may play important roles in pathologic brain network configuration (58, 59), the hypermetabolism in neurodegenerative disorders is often viewed as a controversial finding (60). While hypometabolism is interpreted as reduced synaptic activity, neuronal loss, atrophy, or a combination of these factors, the interpretation of maladaptive hypermetabolism that occurs during disease progression is complex, with numerous explanations (61). It is important to note that our design classified patients at the MCI stage, meaning that increases and decreases in metabolism reflected in the classifier were relative to other patients with PD-MCI and not to healthy controls. As a result, hypermetabolism identified in the PDDCP may reflect only a relatively lesser decrease in metabolism in those regions. Additionally, PDD progression may manifest initially as increased FDG-PET signal in certain regions at the MCI stage. This could result from pathogenic processes associated with neurodegeneration such as neuroinflammation, which has been shown to increase FDG uptake (62, 63). Additionally, increased metabolism heralding a worsening cognitive decline could reflect a compensatory response to PD-related atrophy, in which less affected brain regions show comparative increases in FDG uptake reflecting the additional responsibilities of that region. These explanations are not mutually exclusive, with different interpretations being favored depending on the brain region and disease stage.

Previous studies have identified that increases in FDG-PET signal in the cerebellum, brainstem, white matter, and anterior cingulum are associated with cognitive impairment in PD (24, 56, 57, 64, 65). In our study, increased metabolism was associated with PDD progression in numerous regions including the hippocampus, which has also been reported in AD progression in patients with MCI (25, 66, 67, 68). These findings support the notion that reduced cortical input to the hippocampus may lead to an increase in metabolism, which only shifts to hypometabolism in more advanced stages of the disease when atrophy is more extensive (67–69). In patients with PDD, the hippocampus shows a higher density of Lewy pathology (70–72) and progressive atrophy with disease progression (31, 36, 73–77). A recent meta-analysis of brain imaging studies identified the hippocampus as a central node of an abnormal brain network in PDD, whereas in MCI, this region seems minimally involved (31). This is consistent with patients with PD-MCI who have minimal memory deficits, in contrast to those with PDD, in whom memory is more affected (78). As a result, hypermetabolic nodes such as those found in the hippocampus in our model may be clinically relevant markers of later cognitive decline.

There are several limitations to the current study. We used a relatively small sample size for SVM training, which may have resulted in overfitting. Nevertheless, the k-fold cross-validation showed a good level of sensitivity and specificity (>85%), albeit slightly reduced compared with the results from training. The sensitivity of the SVM classifier was reduced in the independent validation set, although it was not significantly different from the k-fold cross-validation results. It should be noted that there were only 9 MCI-PDD converters in the testing set, therefore, misclassifying only 1 patient would decrease the sensitivity by 11.1%. The sample size of the anti-PD medication cohort was also too small to draw definitive conclusions about the effects of the medication. Therefore, a larger validation study is warranted.

It should also be noted that we did not include any other variables than FDG-PET in the classifier training, to maximize the flexibility of adopting the proposed classifier in any research or clinical setting. Either the resulting labels or the subject scores may be incorporated into a multimodal classifier with other variables, e.g., such as age, sex, symptom severity, genetics, and/or other neuroimaging-based variables, which may improve the accuracy of the overall prediction model (39).

## Methods

### Participants.

In this retrospective chart review study, scans from patients with a clinical diagnosis of PD at the Movement Disorders Clinic at the Asan Medical Center in Seoul, from 2011 to 2015, were used to train the SVM classifier (Table 1). The data were retrieved in March 2018. All patients were diagnosed with PD by a movement disorder specialist according to the United Kingdom Parkinson’s Disease Brain Bank Criteria (79). Decreased dopamine transporter availability was confirmed with 18F-*N*-(3-fluoropropyl)-2β-carboxymethoxy-3β-(4-iodophenyl)nortropane ([^18^F]FPCIT) PET in all patients. The diagnosis of MCI in patients with PD conforms to the MDS PD-MCI criteria (3). All participating patients were assessed with the Addenbrooke’s Cognitive Examination–Revised (ACE-R) as well as the Mini-Mental State Exam (MMSE) (80, 81). The ACE-R comprises 5 cognitive domains: attention/orientation (18 points), memory (26 points), verbal fluency (14 points), language (26 points), and visuospatial ability (16 points), with a maximal attainable score of 100. PD-MCI was determined on the basis of a SD score below normal on at least 2 neuropsychological tests, either as 2 impaired tests within 1 cognitive domain or 1 impaired test in 2 cognitive domains. All patients with PD-MCI showed a gradual cognitive decline that did not significantly interfere with their functional independence. Patients included in the study had a mean follow-up duration of 5 years and underwent FDG-PET imaging and neuropsychological testing upon admission to the study. A PDD diagnosis was determined by a movement disorder specialist on the basis of significant impairment in more than 1 cognitive domain from the premorbid level, with deficits severe enough to impair daily social, occupational, or personal care activities (4). Patients who had symptoms suggestive of atypical Parkinsonism on neurological examination or findings on MRI were excluded. Patients with major depressive disorder, bipolar disorder, schizophrenia, or substance use disorder were also excluded. Patients who later developed dementia during the follow-up period were categorized as converters (PD-MCI/convertors) and those who did not as nonconverters (PD-MCI/nonconverters). Baseline FDG-PET scans from 43 patients, 23 of whom developed PDD and 20 of whom had stable MCI were used for the training of the SVM classifier. For an independent testing set, additional data were retrieved in January 2022 (the baseline FDG-PET scans were taken between 2014 and 2016). In the testing set, a dementia diagnosis was ultimately made clinically by attending neuropsychologists who administered different types of test batteries. If ACE-R was available, it has been included in Table 1. A total of 19 patients were identified in the testing set, 9 of whom were PDD converters, and the remaining 10 patients had stable MCI.

For an additional validation set, FDG-PET scans of 17 patients with PDNC, 19 with PDD, and 18 age-matched healthy controls (NL) were retrieved from Asan Medical Center’s imaging database (Table 1). As an additional validation set, FDG-PET scans of 39 patients admitted to the Crescentwood Memory Clinic in Winnipeg, Manitoba, were used. Among these patients, 22 had clinically diagnosed MCI, and 17 were clinically diagnosed with DLB. DLB is considered to be part of the same spectrum of diseases as PDD and shares similar symptoms and pathological hallmarks (6). A diagnosis of DLB is given if cognitive symptoms precede the development or develop within 1 year of parkinsonian motor symptom onset (5). Additionally, to determine whether the SVM classifier is significantly modulated by anti-PD treatment, an additional 10 patients with clinically diagnosed PD without dementia were scanned at the Health Science Centre in Winnipeg, Manitoba, on separate days, both with their clinically determined anti-PD drug (including l-DOPA) and with their medication withdrawn. Demographic information of the validation sets has been described elsewhere (34, 44).

Patients recruited from the Asan Medical Center were Korean. Ethnicity information was not collected for patients recruited from the Health Science Centre.

### FDG-PET acquisition and preprocessing.

All participants were withdrawn from anti-PD medications for at least 12 hours and fasted for at least 6 hours before scanning. For the patients who were recruited from the Asan Medical Center (Table 1), a 5-minute transmission scan using a ^68^Ge rotating pin source and a 15-minute emission scan were acquired on an ECAT HR+ scanner (Siemens Medical Systems) at the Asan Medical Center, 40 minutes after i.v. injection of 370 MBq FDG (82). For the patients who were recruited from the Crescentwood Memory Clinic and Health Science Centre in Winnipeg (Table 2), all PET imaging data were acquired on a Siemens Biograph 16 HiRez PET/CT (Siemens Medical Solutions) scanner at the University of Manitoba. Patients were injected i.v. with 185 MBq FDG, and a 15-minute static image was acquired starting 40 minutes after injection. A head CT scan was acquired for attenuation correction purposes.

All FDG-PET image preprocessing was carried out using the standard procedure implemented in Statistical Parametric Mapping 12 (SPM) software (www.fil.ion.ucl.ac.uk/spm/). Images were spatially normalized by warping to the Montreal Neurological Institute (MNI) standard space using a PET template and then subsequently smoothed using an 8 mm Gaussian filter. For all images, FDG uptake was proportionally scaled using the whole-brain mean value.

### SVM-based classifier.

SVM model specification was done using the fitcsvm function with linear kerneling implemented in the MATLAB Statistical Toolbox (www.mathworks.com). The ISDA solver was used with the parameters set as follows: outlier fraction = 0.05; no assumption in the initial estimates; misclassification cost = [0 1; 1 0]; tolerance for gradient difference = 0; feasibility gap tolerance = 0; maximal number of numerical optimization iterations = 1,000,000; kernel offset parameter = 0.1; and kernel scale = 1. K-fold cross-validation (k = 10) was performed to evaluate overfitting of the model. The sensitivity and specificity of the k-fold cross-validated model was also calculated.

The SVM model produces a matrix of feature weights that correspond to each voxel. Briefly, we first generated the SVM model based on the training set, which produces a hyperplane that optimally separates the 2 classes. This decision boundary is represented by a set of feature weights (in this case voxel weights) that are dot producted with an individual’s FDG-PET image to produce a subject score. The subject is then classified as either having stable MCI (MCI-MCI) or as a PDD converter (MCI-PDD) on the basis of the sign of the score.

In order to better understand which brain regions were most important in achieving an accurate classification, we used a permutation test to calculate *P* values for the linear predictor coefficients. We built 10,000 SVM models each with randomly permuted class labels, which produced a null distribution of voxel weights. The *P* values were defined as the proportion of times the absolute value of a given voxel weight in the permuted models exceeded the value in the true model. If a given voxel weight had a value greater than the true voxel weight less than 5% of the time, then that voxel could be assigned a value of *P <* 0.05.

### Data availability.

The developed SVM classifier and the scripts used for generating it are available on The Ko Lab’s website: https://www.kolabneuro.com/, and can be found using the search terms “prediction model,” “prognosis,” “support vector machine,” and “biomarker.”

### Statistics.

All statistical analysis was performed using SPSS software for Linux (IBM). Differences in continuous variables (age, disease duration, follow-up period, MDS-UPDRS, MMSE, SVM subject scores) between groups were evaluated using a 2-tailed, 1-way ANOVA (with post hoc Bonferroni’s test) and a 2-tailed Student’s *t* test. Differences in the dichotomic variables (sex and SVM classification labels) between groups were evaluated using a χ^2^ test. A *P* value of less than 0.05 was considered significant.

### Study approval.

Informed consent was acquired from all patients from the Asan Medical Center, the Crescentwood Memory Clinic, and the Health Sciences Center. This study was approved by the research ethics boards of the Asan Medical Center (Seoul, South Korea) and the University of Manitoba (Winnipeg, Canada).

## Author contributions

KWP and CSL performed clinical evaluations of patients and were responsible for providing the brain imaging data. SB performed preprocessing, data and statistical analysis, and prepared the manuscript and figures in consultation with JHK. JHK supervised and conceived of the project. All authors contributed to the revision of the manuscript.

## Supplementary Material

Trial reporting checklists

ICMJE disclosure forms

Supplemental table 1

## Figures and Tables

**Table 2:**
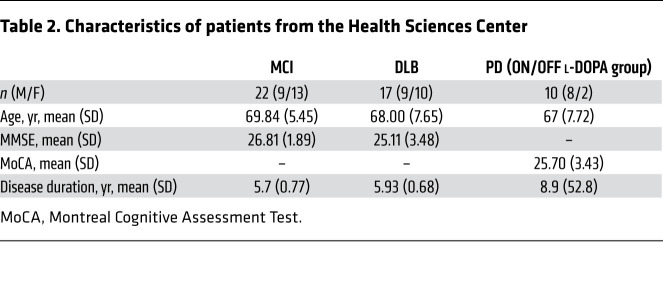
Characteristics of patients from the Health Sciences Center

**Table 1:**
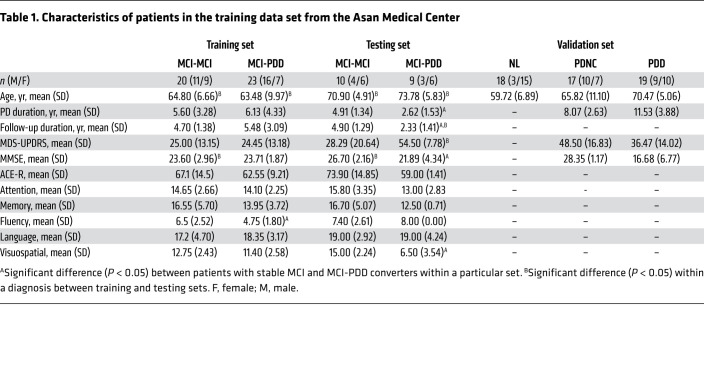
Characteristics of patients in the training data set from the Asan Medical Center

## Version 1 08/30/2022

In-Press Preview

## Version 2 10/17/2022

Electronic publication

## References

[1] Williams-Gray CH (2013). The CamPaIGN study of Parkinson’s disease: 10-year outlook in an incident population-based cohort. J Neurol Neurosurg Psychiatry.

[2] Fredericks D (2017). Parkinson’s disease and Parkinson’s disease psychosis: a perspective on the challenges, treatments, and economic burden. Am J Manag Care.

[3] Litvan I (2012). Diagnostic criteria for mild cognitive impairment in Parkinson’s disease: Movement Disorder Society Task Force guidelines. Mov Disord.

[4] Emre M (2007). Clinical diagnostic criteria for dementia associated with Parkinson’s disease. Mov Disord.

[5] McKeith IG (2017). Diagnosis and management of dementia with Lewy bodies: Fourth consensus report of the DLB Consortium. Neurology.

[6] Jellinger KA, Korczyn AD (2018). Are dementia with Lewy bodies and Parkinson’s disease dementia the same disease?. BMC Med.

[7] Pedersen KF (2013). Prognosis of mild cognitive impairment in early Parkinson disease: the Norwegian ParkWest study. JAMA Neurol.

[8] Domellöf ME (2015). Cognitive function in the early phase of Parkinson’s disease, a five-year follow-up. Acta Neurol Scand.

[9] Goodman D (2013). Prognosis of mild cognitive impairment in early Parkinson disease: The Norwegian ParkWest Study. JAMA J Am Med Assoc.

[10] Hobson P, Meara J (2015). Mild cognitive impairment in Parkinson’s disease and its progression onto dementia: a 16-year outcome evaluation of the Denbighshire cohort. Int J Geriatr Psychiatry.

[11] Morris R (2017). Gait rather than cognition predicts decline in specific cognitive domains in early Parkinson’s disease. J Gerontol A Biol Sci Med Sci.

[12] Lee JE (2018). Association between timed up and go test and future dementia onset. J Gerontol A Biol Sci Med Sci.

[13] Tsiouris KM (2020). Prognostic factors of rapid symptoms progression in patients with newly diagnosed Parkinson’s disease. Artif Intell Med.

[14] Chung SJ (2019). Clinical relevance of amnestic versus non-amnestic mild cognitive impairment subtyping in Parkinson’s disease. Eur J Neurol.

[15] Xu Y (2016). Voxel-based meta-analysis of gray matter volume reductions associated with cognitive impairment in Parkinson’s disease. J Neurol.

[16] Chen PH (2020). Predicting cognitive decline in Parkinson’s disease with mild cognitive impairment: a one-year observational study. Parkinsons Dis.

[17] Yong SW (2007). A comparison of cerebral glucose metabolism in Parkinson’s disease, Parkinson’s disease dementia and dementia with Lewy bodies. Eur J Neurol.

[18] Garcia-Garcia D (2012). Posterior parietooccipital hypometabolism may differentiate mild cognitive impairment from dementia in Parkinson’s disease. Eur J Nucl Med Mol Imaging.

[19] Liepelt I (2009). Cortical hypometabolism assessed by a metabolic ratio in Parkinson’s disease primarily reflects cognitive deterioration-[18F]FDG-PET. Mov Disord.

[20] Jokinen P (2010). [11C]PIB-, [18F]FDG-PET and MRI imaging in patients with Parkinson’s disease with and without dementia. Parkinsonism Relat Disord.

[21] Pagonabarraga J (2013). Spectroscopic changes associated with mild cognitive impairment and dementia in Parkinson’s disease. Dement Geriatr Cogn Disord.

[22] Bohnen N (2011). Cerebral glucose metabolic features of Parkinson disease and incident dementia: longitudinal study. J Nucl Med.

[23] Foo H (2017). Progression of subcortical atrophy in mild Parkinson’s disease and its impact on cognition. Eur J Neurol.

[24] Tang Y (2016). Cerebral metabolic differences associated with cognitive impairment in Parkinson’s disease. PLoS One.

[25] Blum D (2018). Hypermetabolism in the cerebellum and brainstem and cortical hypometabolism are independently associated with cognitive impairment in Parkinson’s disease. Eur J Nucl Med Mol Imaging.

[26] Shoji Y (2014). Neural substrates of cognitive subtypes in Parkinson’s disease: a 3-year longitudinal study. PLoS One.

[27] Baba T (2017). Longitudinal study of cognitive and cerebral metabolic changes in Parkinson’s disease. J Neurol Sci.

[28] Demailly F (2015). Hypometabolism in posterior and temporal areas of the brain is associated with cognitive decline in Parkinson’s disease. J Parkinsons Dis.

[29] Firbank MJ (2017). Cerebral glucose metabolism and cognition in newly diagnosed Parkinson’s disease: ICICLE-PD study. J Neurol Neurosurg Psychiatry.

[30] Weil RS (2019). Neuroimaging in Parkinson’s disease dementia: connecting the dots. Brain Commun.

[31] Mihaescu AS (2019). Brain degeneration in Parkinson’s disease patients with cognitive decline: a coordinate-based meta-analysis. Brain Imaging Behav.

[32] Paulus MP (2019). Machine learning and brain imaging: opportunities and challenges. Trends Neurosci.

[33] Topol EJ (2019). High-performance medicine: the convergence of human and artificial intelligence. Nat Med.

[34] Katako A (2018). Machine learning identified an Alzheimer’s disease-related FDG-PET pattern which is also expressed in Lewy body dementia and Parkinson’s disease dementia. Sci Rep.

[35] Pellegrini E (2018). Machine learning of neuroimaging for assisted diagnosis of cognitive impairment and dementia: a systematic review. Alzheimers Dement (Amst).

[36] Morales DA (2013). Predicting dementia development in Parkinson’s disease using Bayesian network classifiers. Psychiatry Res.

[37] Abós A (2017). Discriminating cognitive status in Parkinson’s disease through functional connectomics and machine learning. Sci Rep.

[38] Ko JH (2014). Quantifying significance of topographical similarities of disease-related brain metabolic patterns. PLoS One.

[39] Grueso S, Viejo-Sobera R (2021). Machine learning methods for predicting progression from mild cognitive impairment to Alzheimer’s disease dementia: a systematic review. Alzheimers Res Ther.

[40] Spasov S (2019). A parameter-efficient deep learning approach to predict conversion from mild cognitive impairment to Alzheimer’s disease. Neuroimage.

[41] Gao Y (2017). Changes of brain structure in Parkinson’s disease patients with mild cognitive impairment analyzed via VBM technology. Neurosci Lett.

[42] Kieburtz K (2006). Issues in neuroprotection clinical trials in Parkinson’s disease. Neurology.

[43] Ko JH (2015). Effects of levodopa on regional cerebral metabolism and blood flow. Mov Disord.

[44] Aljuaid M (2019). Blood flow and glucose metabolism dissociation in the putamen is predictive of levodopa induced dyskinesia in Parkinson’s disease patients. Front Neurol.

[45] Liu AKL (2015). Nucleus basalis of Meynert revisited: anatomy, history and differential involvement in Alzheimer’s and Parkinson’s disease. Acta Neuropathol.

[46] Nakano I, Hirano A (1984). Parkinson’s disease: neuron loss in the nucleus basalis without concomitant Alzheimer’s disease. Ann Neurol.

[47] Jellinger KA (1991). Pathology of Parkinson’s disease. Changes other than the nigrostriatal pathway. Mol Chem Neuropathol.

[48] Shimada H (2009). Mapping of brain acetylcholinesterase alterations in Lewy body disease by PET. Neurology.

[49] Bohnen NI (2015). Frequency of cholinergic and caudate nucleus dopaminergic deficits across the predemented cognitive spectrum of Parkinson disease and evidence of interaction effects. JAMA Neurol.

[50] Hilker R (2005). Dementia in Parkinson disease: functional imaging of cholinergic and dopaminergic pathways. Neurology.

[51] Klein JC (2010). Neurotransmitter changes in dementia with Lewy bodies and Parkinson disease dementia in vivo. Neurology.

[52] Bohnen NI (2003). Cortical cholinergic function is more severely affected in parkinsonian dementia than in Alzheimer disease: an in vivo positron emission tomographic study. Arch Neurol.

[53] van der Zee S (2020). Cholinergic denervation patterns across cognitive domains in Parkinson’s disease. Mov Disord.

[54] Kehagia AA (2012). Cognitive impairment in Parkinson’s disease: the dual syndrome hypothesis. Neurodegener Dis.

[55] Meles SK (2015). Abnormal metabolic pattern associated with cognitive impairment in Parkinson’s disease: a validation study. J Cereb Blood Flow Metab.

[56] Huang C (2007). Metabolic brain networks associated with cognitive function in Parkinson’s disease. Neuroimage.

[57] Huang C (2007). Changes in network activity with the progression of Parkinson’s disease. Brain.

[58] Ko JH (2018). Network structure and function in Parkinson’s disease. Cereb Cortex.

[59] Gupta V, Booth S, Ko JH Hypermetabolic cerebellar connectome in Alzheimer’s disease. Brain Connect.

[60] Borghammer P (2009). Artefactual subcortical hyperperfusion in PET studies normalized to global mean: lessons from Parkinson’s disease. Neuroimage.

[61] Ko JH, Strafella AP (2022). Metabolic imaging and plasticity. Handb Clin Neurol.

[62] Schroeter M (2009). Neuroinflammation extends brain tissue at risk to vital peri-infarct tissue: a double tracer [11C]PK11195- and [18F]FDG-PET study. J Cereb Blood Flow Metab.

[63] Jeong YJ (2017). Assessment of change in glucose metabolism in white matter of amyloid-positive patients with Alzheimer disease using F-18 FDG PET. Medicine (Baltimore).

[64] Tang CC, Eidelberg D (2010). Abnormal metabolic brain networks in Parkinson’s disease from blackboard to bedside. Prog Brain Res.

[65] Apostolova I (2018). Hypermetabolism in the hippocampal formation of cognitively impaired patients indicates detrimental maladaptation. Neurobiol Aging.

[66] Tahmasian M (2015). The lower hippocampus global connectivity, the higher its local metabolism in Alzheimer disease. Neurology.

[67] Das SR (2013). Increased functional connectivity within medial temporal lobe in mild cognitive impairment. Hippocampus.

[68] Pasquini L (2015). Link between hippocampus’ raised local and eased global intrinsic connectivity in AD. Alzheimers Dement.

[69] Harding AJ, Halliday GM (2001). Cortical Lewy body pathology in the diagnosis of dementia. Acta Neuropathol.

[70] Arnold SE (2013). Comparative survey of the topographical distribution of signature molecular lesions in major neurodegenerative diseases. J Comp Neurol.

[71] Hall H (2014). Hippocampal Lewy pathology and cholinergic dysfunction are associated with dementia in Parkinson’s disease. Brain.

[72] Aybek S (2009). Hippocampal atrophy predicts conversion to dementia after STN-DBS in Parkinson’s disease. Parkinsonism Relat Disord.

[73] Weintraub D (2012). Alzheimer’s disease pattern of brain atrophy predicts cognitive decline in Parkinson’s disease. Brain.

[74] Weintraub D (2011). Neurodegeneration across stages of cognitive decline in Parkinson disease. Arch Neurol.

[75] Kandiah N (2014). Hippocampal volume and white matter disease in the prediction of dementia in Parkinson’s disease. Parkinsonism Relat Disord.

[76] Mak E (2015). Baseline and longitudinal grey matter changes in newly diagnosed Parkinson’s disease: ICICLE-PD study. Brain.

[77] Gee M (2017). Regional volumetric change in Parkinson’s disease with cognitive decline. J Neurol Sci.

[78] Noe E (2004). Comparison of dementia with Lewy bodies to Alzheimer’s disease and Parkinson’s disease with dementia. Mov Disord.

[79] Hughes AJ (1992). Accuracy of clinical diagnosis of idiopathic Parkinson’s disease: a clinico-pathological study of 100 cases. J Neurol Neurosurg Psychiatry.

[80] Folstein MF, Folstein SE, McHugh PR (1975). “Mini-mental state”. A practical method for grading the cognitive state of patients for the clinician. J Psychiatr Res.

[81] Mioshi E (2006). The Addenbrooke’s Cognitive Examination Revised (ACE-R): a brief cognitive test battery for dementia screening. Int J Geriatr Psychiatry.

[82] Yoon RG (2015). The utility of susceptibility-weighted imaging for differentiating Parkinsonism-predominant multiple system atrophy from Parkinson’s disease: correlation with 18F-flurodeoxyglucose positron-emission tomography. Neurosci Lett.

